# Urachal adenocarcinoma: a case report and literature review

**DOI:** 10.1093/jscr/rjaf973

**Published:** 2025-12-17

**Authors:** Konstantina Kostara, Lane Palmisano, Pasha Shenasan, Abraham El Sedfy, Derick Christian

**Affiliations:** Department of Surgery, St. Joseph's University Medical Center, 703 Main St., Paterson, NJ 07503, United States; St. George’s University, School of Medicine, Grenada West Indies; Department of Surgery, St. Joseph's University Medical Center, 703 Main St., Paterson, NJ 07503, United States; Department of Surgery, St. Joseph's University Medical Center, 703 Main St., Paterson, NJ 07503, United States; Department of Surgery, St. Joseph's University Medical Center, 703 Main St., Paterson, NJ 07503, United States

**Keywords:** urachus, urachal adenocarcinoma, embryological remnant, umbilicus, urogenital sinus

## Abstract

Urachal carcinoma is a rare malignant epithelial neoplasm with an estimated incidence of one in 5 million people. Due to the rarity of these cases, no universally accepted staging system or standardized surgical and oncological treatment protocols currently exist. En bloc surgical resection of the umbilicus, urachal ligament, and involved portion of the bladder remains the preferred treatment. The addition of adjuvant chemotherapy may be considered in the setting of metastatic disease. Here, we present a case of urachal adenocarcinoma in a 67-year-old male who initially presented with purulent umbilical drainage.

## Introduction

Urachal carcinoma (UrC) is a rare malignant epithelial neoplasm that accounts for 0.01% of all adult cancers [1], with an estimated incidence of one in five million people [2]. UrC constitutes between 0.07% and 0.34% of all bladder neoplasms, the vast majority of which are adenocarcinomas [1–4].

## Case report

A 67-year-old man presented with purulent drainage from the umbilicus. He endorsed unintentional weight loss, and denied any prior screening colonoscopies. Physical exam revealed a palpable suprapubic mass, and laboratory results were notable for anemia. Imaging revealed a large, heterogeneous, centrally necrotic mass arising from the superior aspect of the bladder. The mass extended along the urachus, invading the abdominal wall and sigmoid colon. Central pockets of air within the mass raised concerns for a fistulous tract. Given the findings suggestive of malignancy, a multidisciplinary discussion was held with the consensus for joint en bloc resection by surgical oncology and urology.

Prior to the intra-abdominal portion of the procedure, cystoscopy with ureteral stent placement was performed which revealed a normal urethra, prostatic hyperplasia, and an anterior bladder dome lesion consistent with invasion. Midline laparotomy was performed and the mass was found to be adherent to the anterior abdominal wall, invading the bladder, the posterior rectus sheath, and sigmoid colon. The umbilicus and stalk were excised. The involved sigmoid colon was resected en bloc with the dome of the bladder and associated mass, with grossly clear 5 cm proximal and distal margins. Palpable external iliac lymph nodes were also sent to pathology. Histopathologic evaluation of the surgical specimen demonstrated a moderately differentiated adenocarcinoma, with well-formed, variably sized glands in focal cribriform architecture. Cells stained negative for CK903 and showed a lack of nuclear expression for beta catenin, confirming the diagnosis of urachal adenocarcinoma as opposed to colonic adenocarcinoma. All resection margins were clear, and lymph nodes were negative for metastasis (N0). The patient was discharged with the plan for chemotherapy initiation after full recovery from surgery.

## Discussion

### Origins of the urachus and development of urachal adenocarcinoma

In utero, the fetal bladder develops from the cephalic extension of the urogenital sinus, which connects to a canal-like extension of the yolk sac (the allantois). The connection of these two structures at the level of the umbilicus forms the urachus [5]. As the bladder descends into the pelvis, the urachus stretches and narrows, obliterating the lumen, leaving the urachus as an embryological remnant connecting the dome of the bladder to the umbilicus. This becomes the median umbilical ligament [5].

Histologically, the urachus consists of three layers. The inner epithelial lining is transitional in 70% of cases and columnar in 30%. The submucosal connective tissue layer surrounds the inner epithelial layer, and the outer muscular layer is continuous with the detrusor muscle. When the urachus closes, these layers become indistinct [5].

Urachal carcinoma is a disease of the urachal remnant, 90% of which is adenocarcinoma [5]. It arises in the fifth to sixth decade of life and has a male predominance [5]. The pathogenesis is not fully understood, although it is thought to arise from metaplastic changes in the remnant epithelium [5].

### Clinical features and diagnosis of urachal adenocarcinoma

Given the extraperitoneal location of the urachus, these tumours usually remain undetected until they reach large sizes [2, 3]. Thus, the clinical presentation often remains benign until the tumor invades the bladder, resulting in haematuria. Patients may also experience umbilical pain and discharge, which could be mistakenly diagnosed as infectious in nature [6].

Diagnosis of UrC relies on multiple modalities. Since the neoplasm most frequently involves the anterior wall and dome of the bladder, cystoscopy can be utilized [7]. Findings of a solid or cystic midline bladder mass with micro-calcifications on computed tomography or magnetic resonance imaging are considered pathognomonic [8]. In addition, patients can have elevated CA19-9, CA-125, and carcinoembryonic antigen; levels of these markers may correlate with radiographic responses to chemotherapy [6, 9].

### Staging

There is no current universal staging system for UrC. The 1984 Sheldon staging system is the most referenced staging system. It is based on local invasion and metastasis (Table 1) [2]. The Mayo Clinic staging system is a simplified model that is outcome-based and better correlates with prognosis (Table 2) [6, 7, 10].

**Table 1:** Sheldon staging system for urachal carcinoma (UrC)

Sheldon staging system	
Stage I	No invasion beyond the urachal mucosa
Stage II	Invasion confined to the urachus
Stage III	a) Local extension of UrC into the bladder b) Local extension of UrC into the abdominal wall c) Local extension of UrC into the peritoneum d) Local extension of UrC into viscera other than the bladder
Stage IV	a) Metastases of UrC to the regional lymph modes b) Metastases of UrC to distant sites

Based on the staging described by Sheldon *et al*. [2]

**Table 2:** Mayo staging system for urachal carcinoma (UrC)

Mayo staging system	
Stage I	UrC tumours confined to the urachus and/or bladder
Stage II	UrC tumours that extend beyond the muscular layer of the urachus and/or bladder
Stage III	UrC tumours that infiltrate regional lymph nodes
Stage IV	UrC tumours that infiltrate nonregional lymph nodes or other distant sites

Based on the staging described by Ashley *et al*. [10]

### Treatment options, surgical considerations, and prognosis

Due to the lack of randomized and prospective trials, management of UrC is primarily based on case series and retrospective studies [6, 7]. However, the Canadian Urological Association and Genitourinary Medical Oncologists published a consensus of their treatment recommendations, which consist of en bloc surgical resection of the umbilicus, urachal ligament, and partial cystectomy. In cases where negative bladder margins cannot be achieved with partial cystectomy, a radical cystectomy with en bloc removal of the umbilicus and urachal ligament should be considered [11].

Radiotherapy may be considered for patients who do not qualify for surgery, but the benefits remain unclear. The role of adjuvant chemotherapy is also uncertain and is not routinely recommended [11]. However, in advanced or metastatic cases, FOLFOX is suggested as the preferred regimen, especially if the histology shows features of urothelial carcinoma [11].

Prognosis in UrC seems to be strongly influenced by the stage of the cancer, lymph node involvement, surgical margin status, and tumor grade, and is independent of patient age or gender [4, 6, 7, 11]. Cases with positive lymph node involvement and metastatic disease are associated with worse survival [4, 5].

## Conclusion

Urachal carcinoma is a rare malignancy that arises from the urachus, an embryological remnant that connects the dome of the bladder to the umbilicus. It most commonly presents as adenocarcinoma, likely due to metaplastic changes in the epithelium. Its extravesicular location contributes to its often delayed presentation, and the diagnosis should be considered in patients with atypical presentations and imaging findings of a midline bladder mass. En bloc surgical resection of the umbilicus, urachal ligament, and partial cystectomy remains the preferred treatment for localized disease. A multidisciplinary approach is essential for achieving the best patient outcomes.
